# Diarrhea as a Diagnostic Challenge in Pediatric Appendicitis: A Clinical Cohort and Nationwide Survey of 1,114 Physicians

**DOI:** 10.7759/cureus.115370

**Published:** 2026-08-28

**Authors:** Koji Yokoyama, Mitsukazu Mamada

**Affiliations:** 1 Department of Pediatrics, Japanese Red Cross Wakayama Medical Center, Wakayama, JPN

**Keywords:** clinical decision-making, diagnostic challenge, diarrhea, infectious gastroenteritis, nationwide survey, pediatric appendicitis

## Abstract

Background: Diarrhea may complicate the recognition of pediatric appendicitis because it commonly suggests acute gastroenteritis. We evaluated this diagnostic challenge using a clinical cohort and a nationwide questionnaire survey.

Methods: We retrospectively compared children aged <16 years with acute appendicitis with and without diarrhea at a single tertiary care center. Separately, we conducted a nationwide questionnaire survey of physicians providing outpatient pediatric care in Japan. Distinct items assessed the initial diagnostic impression and intended management in a hypothetical case.

Results: Among 122 children with appendicitis, 21 (17.2%) had diarrhea. C-reactive protein levels were higher in the diarrhea group than in the non-diarrhea group (3.80 vs. 1.65 mg/dL; p=0.006). The perforation rate was numerically higher in children with diarrhea (7/21 (33.3%) vs. 14/101 (13.9%)) but did not reach statistical significance (p=0.067). Among 1,114 physicians who participated in the nationwide survey, 1,035 of 1,110 respondents to the diagnostic item (93.2%) selected acute gastroenteritis as the most likely initial diagnosis. In a separate hypothetical management case, 994 of 1,104 respondents (90%) selected immediate referral.

Conclusions: Diarrhea was not uncommon among children with appendicitis and was associated with higher C-reactive protein levels in this cohort. An initial impression of gastroenteritis was common, while immediate referral was also frequently selected. Persistent or progressive abdominal symptoms should prompt reassessment for appendicitis even when diarrhea is present.

## Introduction

Acute appendicitis is one of the most common surgical emergencies in children, and delayed diagnosis may lead to perforation and prolonged hospitalization [1,2]. Diagnosis can be challenging in pediatric patients because symptoms are often atypical, particularly in younger children [3,4]. Distinguishing appendicitis from acute gastroenteritis is a common clinical problem, especially in outpatient settings where immediate access to imaging may be limited [5]. Pediatric infectious gastroenteritis itself has diverse etiologies and clinical manifestations, including fever, vomiting, abdominal symptoms, and diarrhea [6,7]. Although diarrhea is generally considered suggestive of acute gastroenteritis, it can also occur in children with appendicitis and may complicate the recognition of the condition [8-10].

In our clinical practice, we observed that diarrhea was not uncommon among children with acute appendicitis, prompting us to examine whether this presentation was associated with distinct clinical characteristics. This observation raised a further question: How do physicians at the initial point of care interpret a child presenting with abdominal pain accompanied by diarrhea?

A hospital-based cohort alone cannot address this question. Because diarrhea strongly suggests acute gastroenteritis, it may influence the initial diagnostic impression and potentially obscure the consideration of appendicitis. At the same time, diagnostic impression and management decisions are not necessarily equivalent; a physician may initially favor gastroenteritis while still choosing to refer the patient to exclude a serious alternative diagnosis. Understanding the diagnostic challenge posed by diarrhea therefore requires the evaluation not only of affected patients but also of how physicians perceive and respond to this presentation.

Accordingly, we examined this issue from two complementary perspectives. First, we compared the clinical characteristics and outcomes of children with acute appendicitis with and without diarrhea in a retrospective single-center cohort. Second, we conducted a nationwide questionnaire survey to assess physicians' initial diagnostic impressions and intended management decisions using separate survey items, including a hypothetical management case. A total of 1,114 physicians participated in the nationwide survey. By combining patient-level clinical observations with physician-level diagnostic and management responses, this study aimed to clarify the diagnostic challenges associated with diarrhea in pediatric appendicitis.

## Materials and methods

Study design

This study consisted of two complementary components designed to examine the diagnostic challenge of diarrhea in pediatric appendicitis from different perspectives. The first was a retrospective single-center clinical cohort evaluating the characteristics and outcomes of children with acute appendicitis according to the presence or absence of diarrhea. The second was a nationwide questionnaire survey that assessed physicians' diagnostic impressions and intended management through distinct items, including a hypothetical clinical case. The two components involved independent populations and were analyzed separately.

Clinical cohort

The retrospective clinical cohort was conducted at the Japanese Red Cross Wakayama Medical Center, a regional tertiary referral hospital in Wakayama, Japan. Children aged <16 years who were admitted with acute appendicitis between January 2020 and December 2025 were included. All eligible patients during the study period were consecutively included; patients with recurrent appendicitis and those managed exclusively in outpatient settings were excluded. Clinical information was retrospectively extracted from electronic medical records by one author (KY) and reviewed by the coauthor (MM), including demographic characteristics, gastrointestinal symptoms, laboratory findings, referral status, disease severity indicators, and time from symptom onset to hospital presentation. Laboratory values obtained at admission were used for the analysis. Missing data were infrequent, and no imputation was performed. Acute appendicitis was diagnosed on the basis of imaging findings, surgical pathology, or both. In patients diagnosed by imaging alone, the diagnosis was based on contrast-enhanced computed tomography showing appendiceal enlargement with associated inflammatory changes, as interpreted by a radiologist. No prespecified standardized radiologic cutoff was applied for the purposes of this retrospective study. Surgically treated cases were histopathologically confirmed. Perforation was assessed histopathologically in all surgically treated patients. In patients managed without surgery, perforation status was determined on contrast-enhanced computed tomography by a radiologist. Diarrhea was defined as any documentation of diarrhea from symptom onset to hospital admission. Patients were classified as having diarrhea regardless of stool frequency, consistency, or duration. Diarrhea developing only after admission was not included. Because of the retrospective study design, detailed information regarding stool frequency, consistency, duration, and exact timing was not consistently available. When such details were unavailable, but diarrhea was documented, the patient was still classified as having diarrhea; no cases were excluded solely because these characteristics were missing.

The primary outcomes were perforation and length of hospitalization. Secondary outcomes included inflammatory marker levels and time to presentation.

Nationwide questionnaire survey

A nationwide questionnaire survey was conducted from October 8, 2025, to March 26, 2026, to examine physicians' diagnostic impressions and intended management when encountering a child with symptoms that could represent either acute gastroenteritis or appendicitis. Pediatric outpatient facilities throughout Japan were identified through manual searches of publicly available institutional websites and other electronically accessible sources, with the aim of including pediatric clinics across all 47 prefectures. A paper questionnaire was mailed to 4,274 identified facilities, and one physician providing outpatient pediatric care at each facility was invited to participate. Only one questionnaire was mailed to each identified facility to minimize duplicate responses, and no reminder mailings were sent. Participation was voluntary, respondents could complete the questionnaire anonymously, and no financial or other incentives were provided. Responses were received from 1,114 of the 4,274 contacted facilities (26.1%), with one physician responding per facility.

The questionnaire was developed by the authors specifically for this study and was reviewed for clarity and clinical relevance by the coauthor and several community pediatricians before distribution. No formal pilot testing or validation was performed. The survey collected information on physician characteristics, clinical practice settings, availability of abdominal ultrasonography and blood testing, and clinical findings considered important when evaluating a child with abdominal pain. The questionnaire included separate items assessing physicians' initial diagnostic impressions and intended management. The management component included a hypothetical case describing a five-year-old child with fever, vomiting, right lower quadrant abdominal pain, diarrhea, a C-reactive protein (CRP) level of 7 mg/dL, and a white blood cell count of 12,000/μL in a setting without immediate abdominal ultrasonography. The survey assessed diagnostic impressions and intended clinical actions; it did not measure actual misdiagnosis, referral delays, or patient outcomes. The complete questionnaire, including the diagnostic and management items used in the present analysis, is provided in the Appendices.

Statistical analysis

All statistical analyses were performed using EZR software (Version 1.68; Saitama Medical Center, Kawagoe, Japan) [11]. Continuous variables were expressed as medians with interquartile ranges and compared using the Mann-Whitney U test. Categorical variables were compared using the chi-squared test, with Yates' continuity correction applied when appropriate. A two-sided p-value of <0.05 was considered statistically significant.

Ethical considerations

The study was approved by the Institutional Review Board of the Japanese Red Cross Wakayama Medical Center (approval number: 1554) and was conducted in accordance with the Declaration of Helsinki. An opt-out approach was used for the retrospective clinical cohort. Participation in the nationwide questionnaire survey was voluntary, respondents could complete the questionnaire anonymously, and completion and return of the questionnaire were considered to indicate consent to participate.

## Results

A total of 122 children with acute appendicitis were included. The median age was 10.9 years (interquartile range: 9.0-13.1 years), and 73 (59.8%) were male. Diarrhea was documented in 21 patients (17.2%).

Children with diarrhea had significantly higher CRP levels than those without diarrhea (3.80 (2.24-7.88) vs. 1.65 (0.24-4.30) mg/dL; p=0.006). In an exploratory analysis restricted to patients without perforation, the difference in CRP levels between children with and without diarrhea was attenuated (2.48 vs. 0.90 mg/dL; p=0.051). The perforation rate was higher in the diarrhea group (7/21 (33.3%) vs. 14/101 (13.9%)), although the difference did not reach statistical significance (p=0.067). Time to presentation and length of hospitalization were also numerically longer in the diarrhea group, but the differences were not statistically significant. No significant differences were observed in white blood cell count, fever, or vomiting (Table 1).

**Table 1:** Clinical characteristics of children with acute appendicitis according to the presence of diarrhea Data are presented as median (interquartile range) or number (%). Continuous variables were compared using the Mann-Whitney U test. Categorical variables were compared using the chi-squared test, with Yates' continuity correction applied when appropriate. Test statistics are reported as U or χ^²^ values, as applicable.

Variable	No diarrhea (n=101)	Diarrhea (n=21)	Test statistic	P-value
Time to presentation (days)	2.0 (1.0-2.0)	2.0 (2.0-3.0)	U=804.0	0.066
Length of hospital stay (days)	4.0 (3.0-5.0)	5.0 (4.0-7.0)	U=817.5	0.092
C-reactive protein (mg/dL)	1.65 (0.24-4.30)	3.80 (2.24-7.88)	U=638.0	0.006
White blood cell count (/μL)	14,400 (12,400-16,900)	15,400 (10,500-19,200)	U=1013.5	0.750
Fever, n (%)	62 (61.4%)	16 (76.2%)	χ²=1.65	0.200
Vomiting, n (%)	78 (77.2%)	18 (85.7%)	χ²=0.75	0.390
Perforation, n (%)	14 (13.9%)	7 (33.3%)	χ²=3.36	0.067

Of the 122 children with appendicitis, 114 (93.4%) were referred from other medical facilities. Among these referred patients, appendicitis was suspected at the referring facility in 89 (78.1%). This proportion was similar in children with and without diarrhea.

Nationwide questionnaire survey

Responses were received from 1,114 of the 4,274 contacted facilities (26.1%), with one physician responding per facility. Most respondents, 1,058 (95%), were pediatricians, and 901 (80.9%) had at least 21 years of clinical experience. Abdominal ultrasonography was not routinely available to most respondents: 563 (50.5%) reported no access, and 315 (28.3%) reported limited availability (Table 2).

**Table 2:** Characteristics and clinical practice settings of the 1,114 physicians participating in the nationwide survey Values are presented as number (%), calculated using the total survey population of 1,114 physicians. "Other" includes free-text responses that could not be classified into the prespecified categories. Percentages may not total exactly 100% because of rounding.

Variable	Category	n (%)
Specialty	Pediatrics	1,058 (95)
	General Practice/Internal Medicine	26 (2.3)
	Surgery	8 (0.7)
	Other/unspecified	22 (2)
Years of clinical experience	0-10 years	14 (1.3)
	11-20 years	187 (16.8)
	≥21 years	901 (80.9)
	Missing	12 (1.1)
Primary clinical working hours	Daytime only	720 (64.6)
	Daytime and night/weekend duty	387 (34.7)
	Missing	7 (0.6)
Availability of abdominal ultrasonography	Not available	563 (50.5)
	Limited availability	315 (28.3)
	Routinely available	221 (19.8)
	Other/missing	15 (1.3)
Availability of on-site blood testing (e.g., white blood cell count and C-reactive protein)	Available (same-day results)	961 (86.3)
	Available via external laboratory	106 (9.5)
	Not available	30 (2.7)
	Other/missing	17 (1.5)
Night/weekend clinical coverage	Not available	700 (62.8)
	Partially available	246 (22.1)
	Fully available	87 (7.8)
	Other/missing	81 (7.3)
Use of diagnostic scoring systems or algorithms	Not used	913 (82)
	Pediatric Appendicitis Score (PAS) only	114 (10.2)
	Alvarado/MANTRELS score only	38 (3.4)
	Other or combined responses	39 (3.5)
	Missing	10 (0.9)

**Figure 1:**
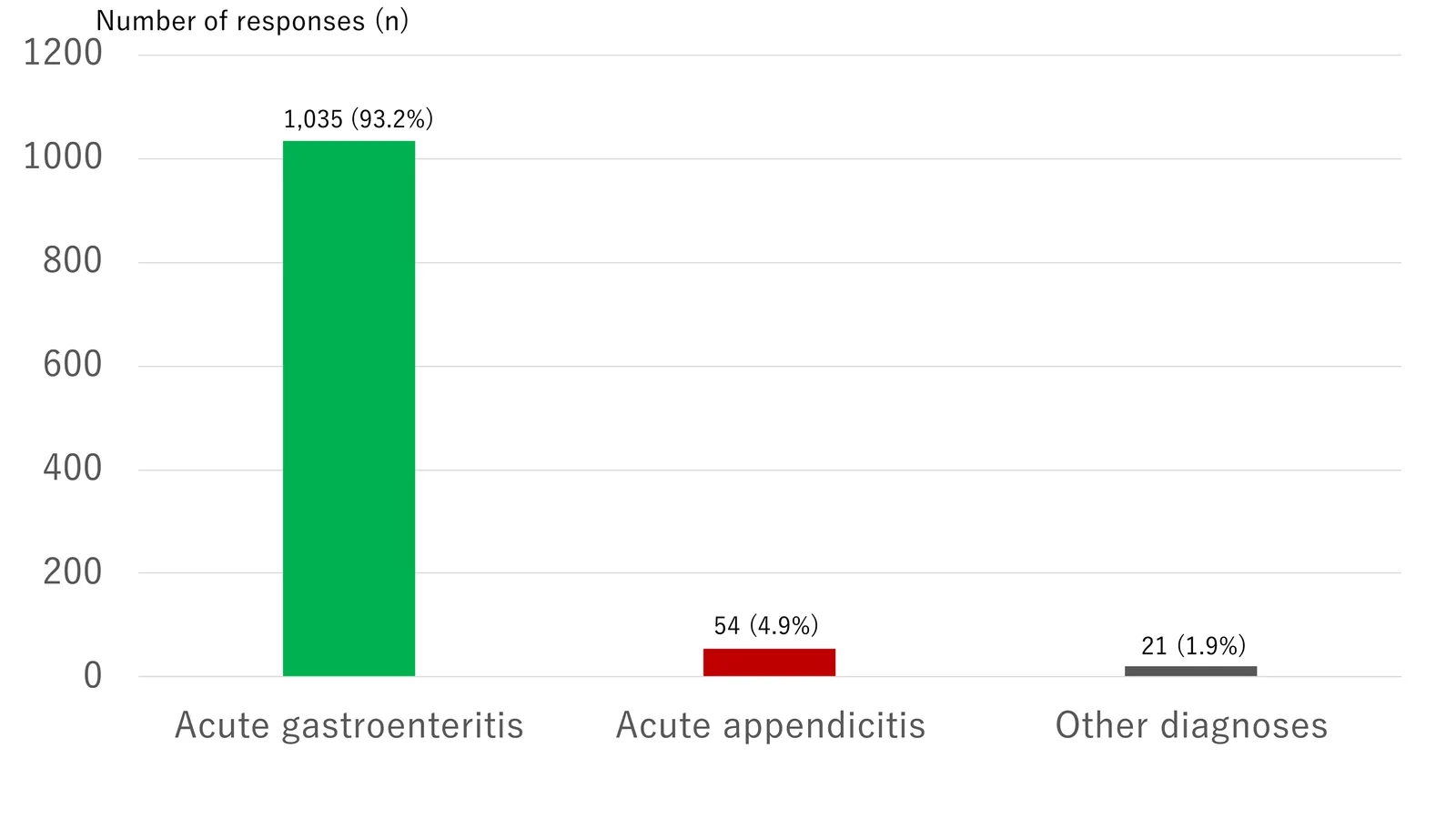
Initial diagnostic impression in the nationwide questionnaire survey Among 1,110 respondents to the diagnostic item, 1,035 (93.2%) selected acute gastroenteritis as the most likely initial diagnosis, whereas 54 (4.9%) selected acute appendicitis, and 21 (1.9%) selected other diagnoses.

In the diagnostic item, 1,035 of 1,110 respondents (93.2%) selected acute gastroenteritis as the most likely initial diagnosis, whereas 54 (4.9%) selected acute appendicitis, and 21 (1.9%) selected other diagnoses (Figure 1).

**Figure 2:**
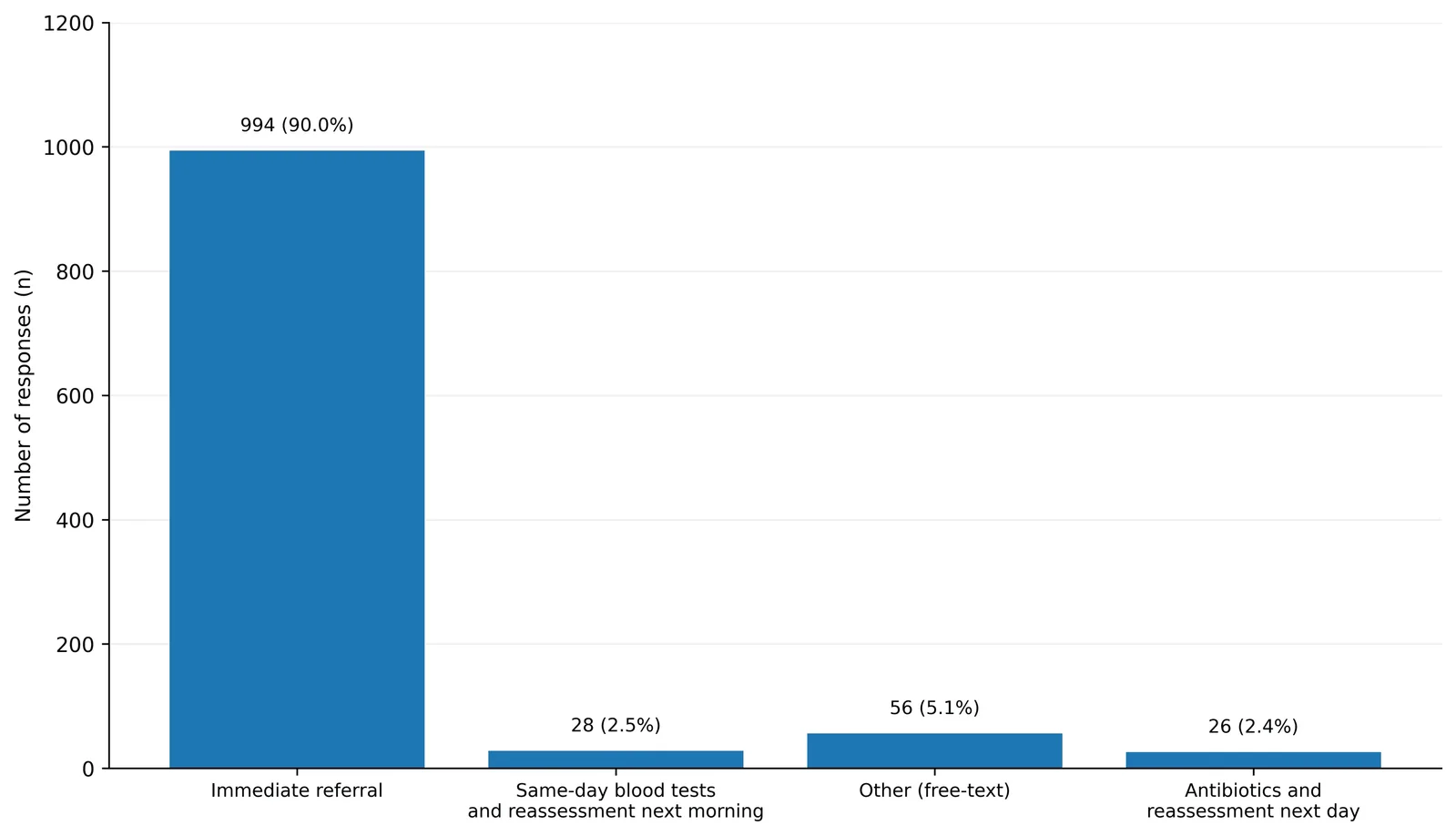
Intended initial management in the nationwide questionnaire survey Among 1,104 respondents to the separate hypothetical management case, 994 (90%) selected immediate referral to a tertiary care center.

In the separate hypothetical management case, 994 of 1,104 respondents (90%) selected immediate referral to a tertiary care center (Figure 2).

**Figure 3:**
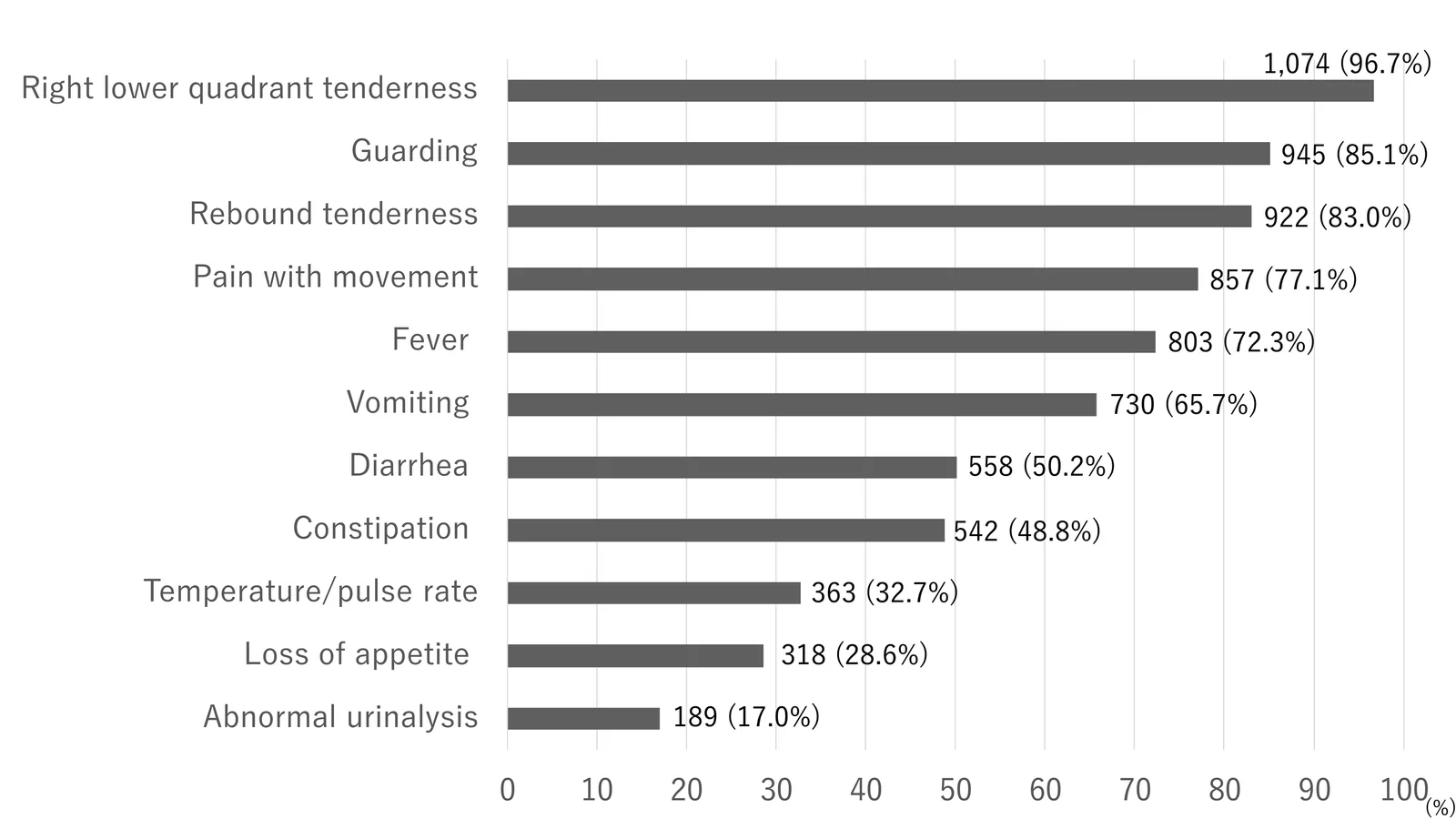
Clinical findings considered important when evaluating a child with abdominal pain Among 1,111 respondents to this item, right lower quadrant tenderness was selected by 1,074 (96.7%), guarding by 945 (85.1%), rebound tenderness by 922 (83%), pain with walking or movement by 857 (77.1%), fever by 803 (72.3%), vomiting by 730 (65.7%), diarrhea by 558 (50.2%), constipation by 542 (48.8%), temperature or pulse rate by 363 (32.7%), loss of appetite by 318 (28.6%), and abnormal urinalysis by 189 (17%). Multiple responses were permitted.

Physicians were also asked which clinical findings they considered important when evaluating a child with abdominal pain. Right lower quadrant tenderness was most frequently selected, followed by guarding, rebound tenderness, and pain with walking or movement (Figure 3).

## Discussion

This study identified two clinically relevant findings. First, diarrhea was present in 21 of 122 children (17.2%) with acute appendicitis and was associated with higher CRP levels. Second, in the nationwide survey of 1,114 physicians, 1,035 of 1,110 respondents (93.2%) to the diagnostic item selected acute gastroenteritis as the most likely initial diagnosis, whereas 994 of 1,104 respondents (90%) selected immediate referral in a separate hypothetical management case.

The nationwide survey provides insight into how physicians interpret this symptom pattern before a definitive diagnosis is established. Children with diarrhea had significantly higher CRP levels than those without diarrhea. However, time to presentation was numerically longer, and perforation was more frequent in the diarrhea group. Furthermore, the difference in CRP levels was attenuated when the analysis was restricted to patients without perforation. These findings suggest that the higher CRP levels observed in children with diarrhea may partly reflect longer disease duration or greater disease severity rather than diarrhea itself. Previous studies have similarly emphasized diarrhea as a potentially misleading presentation of pediatric appendicitis, particularly because gastroenteritis may initially be suspected [8-10]. Naiditch et al. further reported that acute gastroenteritis was the most common initial diagnosis among children with missed appendicitis and missed diagnosis was associated with higher rates of perforation and complications [12]. Horwitz et al. reported the importance of diarrhea as a presenting symptom of appendicitis in very young children [9], while Lu et al. demonstrated substantial clinical overlap between acute appendicitis and gastroenteritis in children presenting with diarrhea [8]. More broadly, delayed recognition of pediatric appendicitis has been associated with an increased risk of perforation and adverse outcomes [1,13,14]. Our findings are consistent with this diagnostic overlap; however, given the small number of patients with diarrhea, the present study cannot establish that diarrhea itself caused delayed diagnosis or greater disease severity. Although acute gastroenteritis was the predominant initial diagnosis in our survey, immediate referral was also frequently selected as the intended management strategy. Thus, an initial impression of gastroenteritis should not necessarily be interpreted as a failure to consider appendicitis or another serious condition.

The characteristics of the respondents may be relevant when interpreting this finding. Most respondents were pediatricians, and 901 (80.9%) had at least 21 years of clinical experience. In addition, routine access to abdominal ultrasonography was reported by only 221 (19.8%) respondents. These characteristics may have influenced referral preferences; however, this interpretation remains speculative because the survey was not designed to identify determinants of individual referral decisions.

The clinical cohort and nationwide survey address different stages of the diagnostic pathway. The cohort describes children after appendicitis has been established, whereas the survey examines physician reasoning before a definitive diagnosis is available. Accordingly, the two datasets should not be linked at the individual level, and the survey cannot explain the higher CRP levels or numerically higher perforation rate observed in the clinical cohort. Rather, they provide complementary information about the clinical presentation of appendicitis with diarrhea and physicians' responses to a similar presentation.

From a clinical perspective, diarrhea should not reduce vigilance for appendicitis when other concerning features are present. Given the potential consequences of delayed diagnosis [1,13,14], persistent or worsening abdominal pain, localization of pain, progressive abdominal findings, or elevated inflammatory markers should prompt reassessment and the consideration of imaging or referral. Importantly, the hypothetical case assessed intended management and did not establish whether respondents would make the same decisions in actual clinical practice.

This study has several limitations. The clinical cohort was retrospective and conducted at a single tertiary referral center, and 93.4% of the patients were referred from other medical facilities. This referral-based population may have preferentially included children with more severe, atypical, or diagnostically challenging presentations. Accordingly, the observed frequency of diarrhea may not directly reflect that among all children with appendicitis presenting to primary or secondary care, and the generalizability of the clinical cohort findings should be interpreted with caution. Only 21 patients had diarrhea, limiting statistical power. Diarrhea was identified from medical record documentation without standardized criteria for stool frequency, consistency, duration, or timing. Patients were classified as having diarrhea whenever it was documented, even when these details were unavailable. Consequently, mild or transient loose stools may have been included, and some degree of exposure misclassification cannot be excluded. Responses were received from 1,114 of the 4,274 contacted facilities (26.1%), and no reminder mailings were sent, raising the possibility of nonresponse bias. Responding physicians may have differed systematically from nonrespondents in their interest in or experience with pediatric abdominal emergencies, although the characteristics of nonrespondents were unavailable for comparison. Therefore, the survey findings may not fully represent pediatric outpatient practice across Japan. The sampling frame was based on publicly available institutional websites and other electronically accessible sources and included facilities from all 47 prefectures; however, some eligible facilities may have been missed, and the distribution of responding facilities was not formally compared with the national distribution of pediatric outpatient facilities. The questionnaire was developed specifically for this study and was not formally pilot-tested or validated, which may limit the reliability and generalizability of the survey measurements. In addition, residual confounding by unmeasured factors, including disease duration and severity, cannot be excluded when interpreting the observed association between diarrhea and inflammatory markers. Furthermore, the survey assessed intended diagnostic impressions and management decisions using questionnaire items and a hypothetical clinical case rather than measuring actual clinical behavior, diagnostic delays, or patient outcomes. Finally, because the clinical cohort and survey involved separate populations, the survey findings cannot be directly linked to the clinical severity observed in the cohort. Despite these limitations, this study combines real-world clinical data with a nationwide survey involving 1,114 physicians from 4,274 contacted facilities. By examining both the clinical presentation and physician decision-making, the study provides a broader perspective on the diagnostic challenge posed by diarrhea in pediatric appendicitis than either approach alone.

## Conclusions

Diarrhea was not uncommon among children with acute appendicitis and was associated with higher CRP levels in our clinical cohort. In the nationwide survey, acute gastroenteritis was the predominant initial diagnostic impression, although most respondents selected immediate referral. Diarrhea should not be considered sufficient to exclude appendicitis, and persistent or progressive abdominal symptoms may warrant reassessment and the consideration of imaging or referral.

## Appendices

**Table 3:** Author-developed questionnaire used in the nationwide survey The questionnaire was developed by the authors specifically for this study and was originally administered in Japanese. The English version below was translated from the original Japanese questionnaire by the authors and reviewed by the coauthor. No copyrighted or licensed questionnaire, scale, or instrument was used. Respondents could answer anonymously, and providing the institution and respondent names was optional. These identifying-information fields were not used in the present analysis.

Item	Question and response options
1	Institution and respondent name: Optional free-text response (Respondents could leave these fields blank.)
2	Medical specialty: Pediatrics/Internal Medicine/General Practice/Other
3	Years of clinical experience: 0-5 years/6-10 years/11-20 years/≥21 years/Other
4	Primary clinical working hours: Daytime only/Daytime plus night and holiday duty
5	Availability of abdominal ultrasonography at the respondent's institution: Routinely available/Available on a limited basis/Not available/Other
6	Availability of blood testing (e.g., white blood cell count and C-reactive protein) at the respondent's institution: Same-day results available/Sent to an external laboratory/Not available/Other
7	Nighttime and holiday clinical coverage: Available/Partially available/Not available/Other
8	Clinical findings considered important when evaluating a child with abdominal pain (multiple responses allowed): Fever/Right lower quadrant tenderness/Rebound tenderness/Pain with walking or movement/Loss of appetite/Vomiting/Diarrhea/Constipation/Temperature or pulse rate/Guarding on palpation/Abnormal urinalysis/Other
9	Use of diagnostic scores or algorithms (multiple responses allowed): Pediatric Appendicitis Score (PAS)/Pediatric Appendicitis Risk Calculator (pARC)/Alvarado score or MANTRELS score/Not used/Other
10	Use of C-reactive protein in the initial assessment: Used/Used depending on the case/Not used/Other
11	If C-reactive protein is used, approximate C-reactive protein level at which hospitalization or referral would be strongly considered: Free-text response (mg/dL)
12	Preferred approach to imaging when abdominal ultrasonography is unavailable at the respondent's institution: Referral for ultrasonography/Referral for CT evaluation/Observation and reassessment/Other
13	Initial management after a diagnosis of acute appendicitis before referral (multiple responses allowed): Restriction of oral intake/Fluid therapy/Analgesia/Antimicrobial therapy/Imaging request/Immediate referral/Other
14	Factors prompting immediate referral (multiple responses allowed): Signs of peritoneal irritation/Persistent high fever/Elevated white blood cell count/Elevated C-reactive protein/Suspected appendiceal enlargement on imaging/Prolonged symptoms/Younger age/Repeated visits/Strong parental concern/Other
15	Approximate time from presentation to referral: Same day/Within 24 hours/Within 2-3 days/Depending on the case/Other
16	Information expected from the referral hospital (multiple responses allowed): Initial imaging interpretation/C-reactive protein and white blood cell count trends/Decision regarding the need for surgery/Conservative treatment plan/Criteria for reassessment/Explanatory materials for the family/Other
17	Would diagnostic feedback after referral be useful? Very useful/Useful/Neither/Not useful/Other
18	Preferred format for feedback after referral (multiple responses allowed): Referral reply letter/Regional medical meeting/Other
19	For a child with abdominal pain and diarrhea, which diagnosis would you prioritize at the initial visit? Respondents were instructed to assume a child aged 3-12 years, symptom onset within 48 hours, a temperature of approximately 38°C, and no blood testing or ultrasonography performed at the time of assessment and to select the single diagnosis they would prioritize first: Acute gastroenteritis (primarily presumed viral)/Bacterial enteritis (including foodborne illness)/Acute appendicitis (including appendicitis presenting with diarrhea)/Constipation-related disorder (e.g., overflow diarrhea)/Lactose intolerance or food allergy-related disorder/Urinary tract infection/Other
20	Hypothetical case: A five-year-old child with fever, vomiting, right lower quadrant abdominal pain, diarrhea, C-reactive protein 7 mg/dL, white blood cell count 12,000/μL, and no ultrasonography available. Initial management: Immediate referral/Additional blood testing on the same day and reassessment the following morning/Antimicrobial treatment and reassessment the following day/Other (free-text response)
21	Free-text comments
